# Deep Inspiration and the Emergence of Ventilation Defects during Bronchoconstriction: A Computational Study

**DOI:** 10.1371/journal.pone.0112443

**Published:** 2014-11-17

**Authors:** Amir H. Golnabi, R. Scott Harris, Jose G. Venegas, Tilo Winkler

**Affiliations:** 1 Department of Anesthesia, Critical Care and Pain Medicine, Massachusetts General Hospital and Harvard Medical School, Boston, Massachusetts, United States of America; 2 Department of Medicine, Pulmonary and Critical Care Unit, Massachusetts General Hospital and Harvard Medical School, Boston, Massachusetts, United States of America; Technion - Israel Institute of Technology, Israel

## Abstract

Deep inspirations (DIs) have a dilatory effect on airway smooth muscle (ASM) that helps to prevent or reduce more severe bronchoconstriction in healthy individuals. However, this bronchodilation appears to fail in some asthmatic patients or under certain conditions, and the reason is unclear. Additionally, quantitative effects of the frequency and magnitude of DIs on bronchodilation are not well understood. In the present study, we used a computational model of bronchoconstriction to study the effects of DI volumes, time intervals between intermittent DIs, relative speed of ASM constriction, and ASM activation on bronchoconstriction and the emergence of ventilation defects (VDefs). Our results showed a synergistic effect between the volume of DIs and the time intervals between them on bronchoconstriction and VDefs. There was a domain of conditions with sufficiently large volumes of DIs and short time intervals between them to prevent VDefs. Among conditions without VDefs, larger volumes of DIs resulted in greater airway dilation. Similarly, the time interval between DIs, during which the activated ASM re-constricts, affected the amplitude of periodic changes in airway radii. Both the relative speed of ASM constriction and ASM activation affected what volume of DIs and what time interval between them could prevent the emergence of VDefs. In conclusion, quantitative characteristics of DIs, such as their volume and time interval between them, affect bronchoconstriction and may contribute to difficulties in asthma. Better understanding of the quantitative aspects of DIs may result in novel or improved therapeutic approaches.

## Introduction

Deep inspirations (DIs) can be an effective bronchodilator and protect against bronchoconstriction. But these beneficial effects are reduced or completely absent in patients with asthma, and the reasons for that are still unclear. Largely unknown are the effects of DIs on the heterogeneity in airway narrowing during bronchoconstriction that causes patchy ventilation defects (VDefs). DIs may not affect all airways equally when VDefs are present resulting in differences in dilation among airways that affect observations of global lung behavior.

Imaging studies have shown the formation of VDefs during bronchoconstriction as a result of severe narrowing or occlusion of airways, e.g. [1]–[4]. Strikingly, a computational integrative model of bronchoconstriction was able to explain the emergence of such pattern of ventilation consistent with imaging results [1]. That model was based on an airway tree network where interactions among the airways of the tree led to the emergence of heterogeneous constriction and VDefs. Such emergent behavior in complex systems is linked to critical points or boundaries between domains with very different behaviors [5]. At a critical point, complex systems are very sensitive to external stimuli so that DIs may prevent, eliminate or reduce VDefs, or not change them at all if the system is far away from a critical point. In fact, Tzeng et al. have demonstrated in an imaging study that DIs may reduce ventilation heterogeneity with an effect that appeared to be smaller in asthmatics compared to non-asthmatics [6]. In addition, studies using global measures of pulmonary function have demonstrated that DIs with volumes lower than inspiratory capacity (IC) resulted in smaller changes in airway resistance [7], specific airway conductance [8], airway lumen area [9], residual volume, and partial expiratory flow [10]. Although several studies using a single DI or sequences of multiple DIs have shown reductions in airway resistance in bronchoconstricted non-asthmatic subjects [11]–[13], it is still unclear how the individual airways of a heterogeneously constricted bronchial tree respond to DIs.

Experimental evidence supporting the bronchodilating effect of DIs includes the effects of: static and dynamic loads applied to smooth muscle strips [14]–[16], differences in lung volume [17], changes in tidal volume [18], and changes in tidal stretch amplitude [14], [19]. An elegant concept unifying static and dynamic effects on airway smooth muscle was first incorporated as peak transmural pressure in a mathematical model of a single terminal airway [20]. It is important to note that this transmural pressure included not only the pressure difference across the airway wall, but was also affected by the parenchymal forces caused by both lung inflation and parenchymal distortion during bronchoconstriction. For the bronchial tree in our model, that single airway model was incorporated in each individual airway [1], and affected the feedback mechanism responsible for the emergence of VDefs during bronchoconstriction [1], [5]. Given that DIs in lungs with VDefs may lead to regionally different peak transmural pressures, the response to DIs may be different among airways.

During spontaneous breathing, sighs (or equivalently DIs) occur fairly periodically [21], and they are thought to have a bronchoprotective effect similar to that seen in studies of agonist stimulations [10], [22], [23]. However, depending on the baseline level of smooth muscle tone, slow re-constriction is usually observed following a DI [24], which suggests that not only the volume of DIs, but also the time interval between them may be very important. The literature on DIs is complicated, as DI characteristics are often different among studies, including for example DIs reaching approximately total lung capacity (TLC) [25]–[27], or DIs of only two to three times the tidal volume [13], [28]. Statistics of the volume and frequency of spontaneous DIs are sparce, making estimating the dilating effect of spontaneous DIs on airway smooth muscle difficult. Boulet et al. published for example an interesting cumulative frequency distribution of tidal breath volumes, but only for the range from 100 to 200% of the mean tidal volume [29].

In this computational study, we hypothesized that responses in airway resistance to DIs observed in human subjects [1] could be replicated with our integrative model of bronchoconstriction. Furthermore we aimed at insights into the effects of DIs characteristics on the emergence of heterogeneous bronchoconstriction and VDefs that would be difficult to measure experimentally because of the complex behavior of the airways. Our objective was to evaluate how DI volume and the time interval between DIs, prevent the emergence of VDefs, and what DI characteristics fail to prevent the emergence of VDefs. We discovered that the computational model demonstrated some intuitive findings that fit with clinical data such as the synergistic nature of increasing volume and frequency of DI's on reducing VDefs, but that the model also revealed counter-intuitive findings such as the worsening of VDefs with insufficient volume or frequency of DIs and the ability to predict the efficacy of DI's based on a single parameter derived from the rate of airway smooth muscle contraction.

## Methods

### Computational Model

An integrative computational model of bronchoconstriction, previously described in detail [1], was employed for our simulation studies. Briefly, this model incorporates the relationships for a single airway model proposed by Anafi and Wilson [20] into each of the 8191 airways of a bronchial tree with 12 generations. Airflows and pressures throughout the bronchial tree and volume changes in the terminal units were calculated with time steps of 10 milliseconds (ms). Airway radii were constant during each breathing cycle, but they were updated from breath to breath in response to each airway's peak transmural pressure during the preceding breathing cycle. As a result, the behavior of each airway depended on the forces acting on its wall, but its constriction affected and was affected by the distribution of airflows and pressures throughout the bronchial tree and the regional expansion in the lungs during breathing. Additionally, we implemented a recursive function with speed indexes of constriction (*S_c_*) and dilation (*S_d_*) that characterize the dynamics in the response of airway radii. If the current radius of an airway is different from its predicted steady state radius for the current breath then *S*_c_ or *S*_d_ determine the fraction by which the airway radius changes from breath to breath from its current radius towards the predicted radius. The recursive function is defined as: *r*_i_(j,k+1)  = *r*_i_(j,k) +*S*_x_ * (*r* ^*^_i_(j,k) – *r*_i_(j,k)) with *S*_x_ = *S*_c_ for (*r* ^*^_i_(j,k) – *r*_i_(j,k)) <0, and *S*_x_ = *S*_d_ for (*r* ^*^_i_(j,k) – *r*_i_(j,k))>0, where *r*_i_ is the inner radius of airway j at the current breath k or the following breath k+1, and r^*^_i_ is the predicted steady state value for the conditions of breath k. In principle, the recursive function is an implementation of a time constant for the discrete time steps of the breathing cycles. Estimates from published experimental data of airway lumen [30] and airway smooth muscle [14] yielded speed indexes of constriction (*S_c_* = 0.05) and dilation (*S_d_* = 0.33) for 12 breaths per minute. The hierarchical network of the bronchial tree defines the interconnections among individual airways – each having local interactions between smooth muscle and airway wall mechanics. Length and diameter of fully dilated airways were defined according to Weibel's morphometric data for the human bronchial tree. The computational model was solved iteratively using MATLAB (MathWorks, Natick, MA).

### DI Characteristics

**Figure 1:**
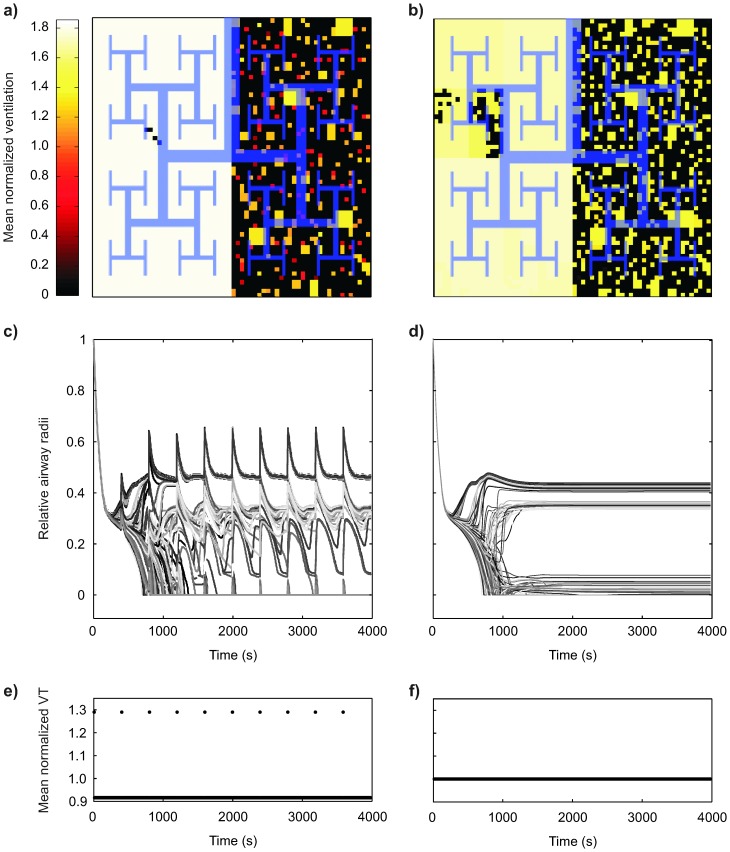
Examples of two-dimensional maps of ventilation distribution, and dynamics of relative airway radii with the corresponding breath-by-breath tidal volumes. The ventilation maps show differences between breathing conditions with DIs (a) and without (b) on the regional distribution of ventilation among the 4096 terminal units of the model using a 64×64 grid. The overlaid schematic tree illustrates the connectivity among the terminal units with a Mandelbrot-like tree structure for the six most central airway generations of the model that has in total 12 generations of bifurcations. Each perpendicular bisector corresponds to a bifurcation point in the bronchial tree. Also, the ventilation of each terminal unit corresponds to a specific pixel in the ventilation map and the color scale corresponds to the mean normalized ventilation, i.e. ventilation normalized by the average ventilation during the simulation. The relative airway radii of 256 randomly selected terminal airways over a time course of 4000 s (c) with and (d) without DIs show the response to the corresponding breath-by-breath tidal volumes (e) with and (f) without DIs. The example with DIs shows a DI cycle of 400 s including one DI followed by 79 normal tidal breaths, each of the 80 breaths was 5 s long. V_DI_/V_T_ was 1.4 and the mean tidal volume was adjusted so that all compared conditions would provide the same ventilation to an individual.

Effects of the DI characteristics on the pattern of bronchoconstriction and the emergence of VDefs were evaluated for a range of different periodic DIs. The DIs were characterized by their relative volume and the time interval of their periodic cycles (or equivalently, the frequency of DIs), including one DI followed by multiple breaths with normal tidal volume before proceeding to the next DI. The breathing frequency was 12 breaths per minute, so that all time intervals of DI cycles were multiples of 5 seconds (s), see for example Fig. 1e. We investigated the time intervals of DIs cycles of 25, 50, 100, 200, 400, 800, and 1600 s, which includes values below and above the average 10 sighs per hour reported in the literature [21] that are equal to an average cycle of 360 s. The relative volume of DIs was defined as the ratio between the volume of the DI and the tidal volume of breaths between them (V_DI_/V_T_). Simulation of conditions with combinations of different time intervals of DI cycles and relative volume of DIs were conducted in two groups. One group focused on shorter time intervals (25, 50, 100, 200, and 400 s) in combination with smaller V_DI_/V_T_ (1.2, 1.4, …, 3.0), while the other group focused on longer time intervals (400, 800, 1600 s) and larger relative volumes of DIs (3, 4, 5, 6), where we suspected that the response to DIs may be substantially different from the lower parameter ranges. It should be noted that the relative DI volume of 6.0 corresponds approximately to inspiratory capacity. Each breathing cycle consisted of a constant inspiratory flow and a passive exhalation to a positive end-expiratory pressure (PEEP) of 5 cmH_2_O. To compare conditions with the same mean ventilation, we adjusted the tidal volume of the breaths between DIs. For each condition, we simulated 4000 s (800 breaths) to allow the numerical model to reach stable conditions of bronchoconstriction. To explore a potential link between *S_c_* and the time interval between DIs, we doubled the speed index of constriction (*S_c_* = 0.1) and repeated the set of conditions defined above.

### Comparison with Experimental Data

For comparison of the global behavior of the model with experimental data, we computed global airway resistance (*R*) as the real part of the impedance at 6 Hz, since this is a frequency commonly used for the forced oscillation technique. Equivalent to the numerical model, terminal units were assumed to be compliances with an impedance of – *i*/*ωC*, where *ω* is the angular frequency and *C* is the capacitance, and airways were assumed to have a linear resistance as described by Poiseuille's flow using the radii and lengths of the constricted airways from the numerical simulation. For comparison with experimental data from Hulme et al [7], we selected a specific DI characteristic that had a drop in relative resistance similar to the experimental data (relative volume of DI of 3.0 and time interval of DI cycles of 100 s). We computed the relative breath-by-breath resistance as a percentage of the resistance at the breath prior to the DI, i.e. pre-DI resistance (*R*_Pre-DI_). To further compare our simulation results with experimental data from Hulme et al [7], we calculated *R*_Pre-DI_ as well as *R*_Post-DI_ (post-DI resistance at the breath following the DI), for the range of V_DI_/V_T_ = 1.2 to 3.0 with a time interval of DI cycles of 200 s. Then, we normalized these values by the airway resistance of the unconstricted airway tree, *R_0_*. For comparison of the model's characteristic responses with experimental data, we used the normalized response in *R*_Post-DI_ calculated as *R*_Post-DI_^*^  =  (*R*_Post-DI_ – *R*_min_)/(*R*_Pre-DI_ – *R*_min_), where *R*_min_ was the minimum of the set of *R*_Post-DI_ values, both for simulated and experimental data. Additionally, we calculated the percentage of relative post-DI resistance (*R*_Post-DI_/*R*_Pre-DI_).

### Estimated Parameters and Data Visualization

Regional ventilation maps along with an index representing the fraction of closed terminal units (*F_c_*) for all DI combinations were calculated for each individual breath. Terminal units were considered as “closed” or severely hypoventilated when they received less than 15% of the average ventilation per terminal unit. *F_c,mean_* was defined as the mean value of *F_c_* over all breaths within the a complete DI cycle. The distribution of regional ventilation is presented with maps using a 64×64 grid for the 4096 terminal units, interconnected by a 12 generations Mandelbrot-like tree (Fig. 1a). There, the ventilation of each terminal unit corresponds to a specific grid element in the ventilation map. The Mandelbrot-like tree defines also how closely related the mapped elements are in the branching hierarchy of the bronchial tree. The color scale corresponds to the mean-normalized local ventilation. All ventilation maps presented in the paper have the same color scale as that in Fig. 1a. Finally, for each airway a relative luminal radius (*r/r_0_*) was defined as the ratio of luminal radius (*r*) normalized by the corresponding value for full dilation (*r_0_*). For visualization purposes, plots showing the evolution of relative radii over time (4000 s) were generated for 256 randomly selected terminal airways (out of the 4096), as illustrated in Fig. 1c.

### Random Perturbations

In order to perturb the unstable equilibrium of a perfectly symmetric tree, a small random heterogeneity (1% coefficient of variation) was added to the wall thickness of all airways. To investigate the effect that different realizations of the random perturbations could have had on the emergence of VDefs and on *F_c,mean_* values, we conducted simulations using 10 different random realizations of the perturbations in wall thickness for each set of the DI characteristics described above. The range and the average of *F_c,mean_* values from the 10 random realizations were calculated.

### Level of Smooth Muscle Activation

Simulations with different levels of airway smooth muscle activation were conducted using relative smooth muscle activation factors (*T_r_*) of 1.0, for the maximal tension that airway smooth muscle generates during nearly full activation [17], [20], and of 0.9, for 90% of maximum activation. All simulations described above were performed at *T_r_* = 1.0, and for the group of conditions with shorter time intervals of DIs cycles (25, 50, 100, 200, and 400 s) and smaller relative volumes of DIs (1.2, 1.4, …, 3.0), they were repeated at *T_r_* = 0.9 using one of the random realizations.

## Results

### Model Predictions of Global Airway Resistance vs. Experimental Data

**Figure 2:**
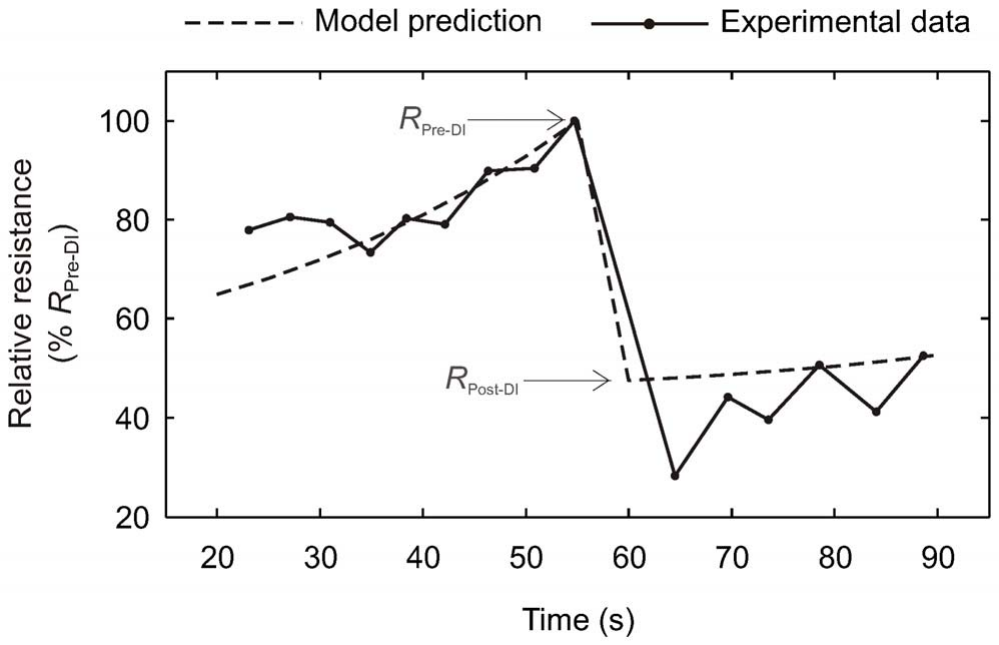
Comparison of computational model prediction of relative post-DI airway resistance (*R*_Post-DI_/*R*_Pre-DI_) with experimental data. The selection of breaths for pre- and post-DI resistance values is marked.

The relative change in global airway resistance (% *R*_Pre-DI_) in response to a single DI in the model for V_DI_/V_T_ = 3.0 and a time interval of DI cycle of 100 s was similar to the change of the normalized resistance of the respiratory system in the experimental study by Hulme et al (Fig 2). The relative resistance dropped to 48% in the model compared to 28% in the example of experimental data. Moreover, the time course of global resistance prior to and after the DI in the model appears to follow well the experimental data.

**Figure 3:**
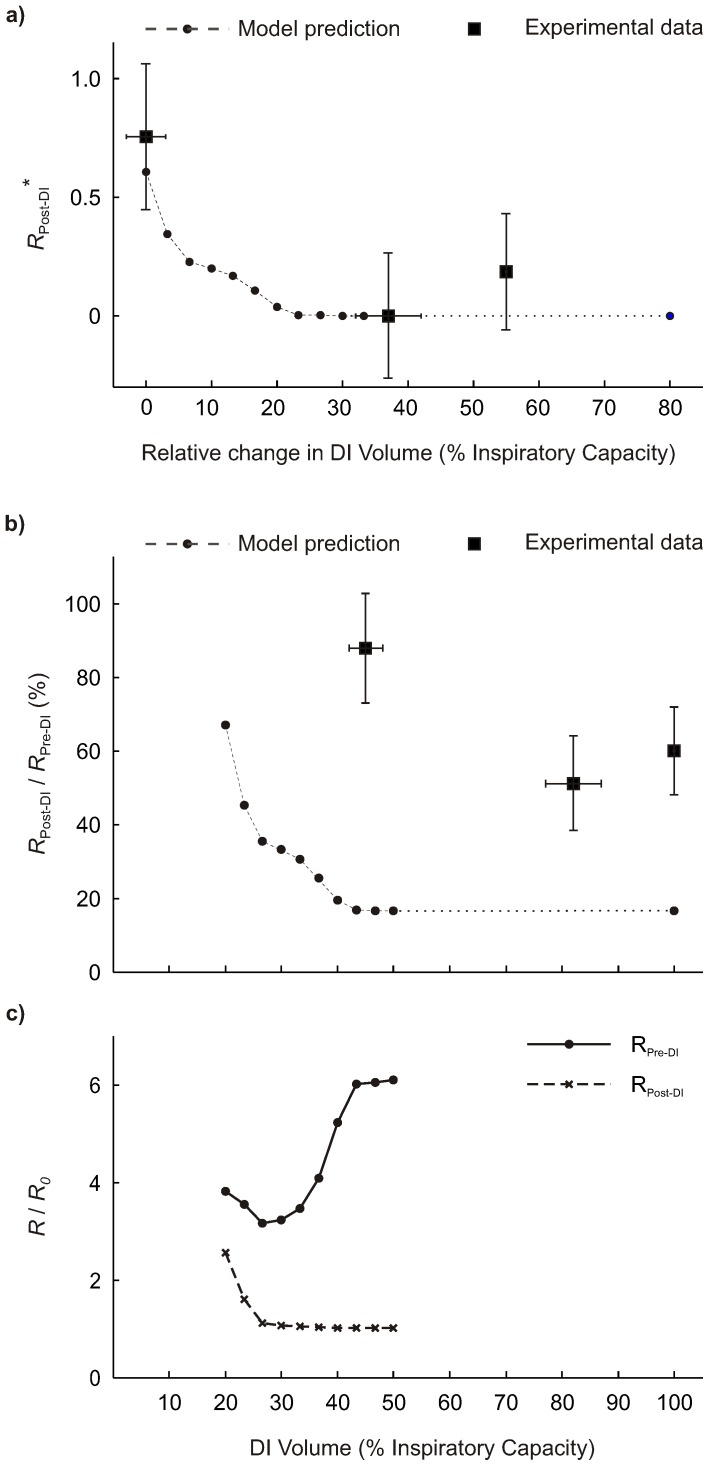
Comparison between model prediction and experimental data from (Hulme et al) for the effect of DI volume on the response in global airway resistance parameters. (a) Normalized response in *R*_Post-DI_* as a function of relative change in DI volume (% inspiratory capacity, IC), for simulated (dashed line) and experimental data (displayed as mean ±95% CI). *R*_Post-DI_^*^  =  (*R*_Post-DI_ – *R*_min_)/(*R*_Pre-DI_ – *R*_min_), where *R*_min_ values of the predicted and experimental data were the minimum of the corresponding set of *R*_Post-DI_ values; (b) Relative post-DI resistance (*R*_Post-DI_ */R*_Pre-DI_) of experimental data (displayed as mean ±95% CI) and computational model prediction (dashed line); (c) Model prediction of pre- (solid) and post-DI (dashed) airway resistance normalized by that of the fully dilated airway tree (*R*/*R_0_*). For the plots in b and c showing DI volume relative to IC, we assumed that IC = 3600 ml, so that a full IC maneuver corresponds to V_DI_/V_T_ = 6 in the model.

Comparison of responses to different DI volumes showed that the model's prediction of *R*_Post-DI_^*^ follows closely the characteristic differences among the few data points of the experimental data (Fig. 3a). For the range from the lowest DI volume – approximately equal to the onset of responses in *R*_Post-DI_^*^– to 30% above the onset, larger DI volumes resulted in lower *R*_Post-DI_^*^. Above that range, the model predictions were virtually identical because DIs fully dilated the airways. (dotted line), which is consistent with the non-significant difference between experimental data points of that range. Also, we found differences between the model's prediction and experimental data in *R*_Post-DI_/*R*_Pre-DI_ that had no effect on the characteristics of responses to different DI volumes described above (Fig. 3b). The model's prediction of the response in *R*_Pre-DI_ and *R*_Post-DI_ to different DI volumes showed that the changes in both parameters are relevant for the interpretation of changes in *R*_Post-DI_/*R*_Pre-DI_ (Fig. 3c); as the DI volume increased, *R*_Post-DI_ declined until it reached the limit of full dilation of the airways (*R*_Post-DI_/*R*_0_ = 1). Interestingly, *R*_Pre-DI_ was not constant over the tested DI volumes but showed a non-monotonous response that is associated with the periodic DIs used in our study, the affect of DI volume on the fraction of VDefs and the level of narrowing of airways outside of VDefs. This finding suggests that the breathing history prior to a DI may affect the magnitude of the change in *R*_Post-DI_/*R*_Pre-DI_ in experimental studies.

### DIs and the Heterogeneity of Bronchoconstriction

Interactions between DIs and local bronchoconstriction occurred during and after the emergence of VDefs and resulted in substantial differences in airway behavior compared to monotonous breathing without DIs, but equivalent mean ventilation (Fig. 1). The ventilation maps for periodic DIs with a volume of DIs of 1.4 and a time interval of the DI cycle of 400 s compared to monotonous tidal breathing without DIs showed an example of such interactions; DIs resulted in a different pattern of VDefs and in more poorly ventilated terminal units, as the larger number of dark colored units inside the VDefs illustrated (Fig. 1a compared to 1b). The brighter color outside of the VDefs indicate increased regional ventilation, which is consistent with a redistribution of ventilation from the inside to the outside of the VDefs compared to the ventilation map for breathing without DIs. Changes in airway radii during the early phase of the emergence of heterogeneity in bronchoconstriction showed that the DI at 400 s did not homogenize the airway radii, but rather caused an inhomogeneous partial dilation in all airways without reversing or stopping the increase in heterogeneity among airways (Fig 1c and 1d). Subsequent DIs clearly made the constriction more heterogeneous causing dilation in the least constricted airways compared to tidal breathing without DIs, but increased constriction and hypoventilation inside of VDefs (Fig. 1). Later during the stable state bronchoconstriction (last 500 s in Fig. 1c), DIs dilated the least constricted airways and those that were not completely closed, but had virtually no effect on closed airways. These differences between a breathing pattern with and without DIs demonstrate that DIs interact at a local level with the heterogeneity in bronchoconstriction.

### DI Characteristics and Airway Behavior

**Figure 4:**
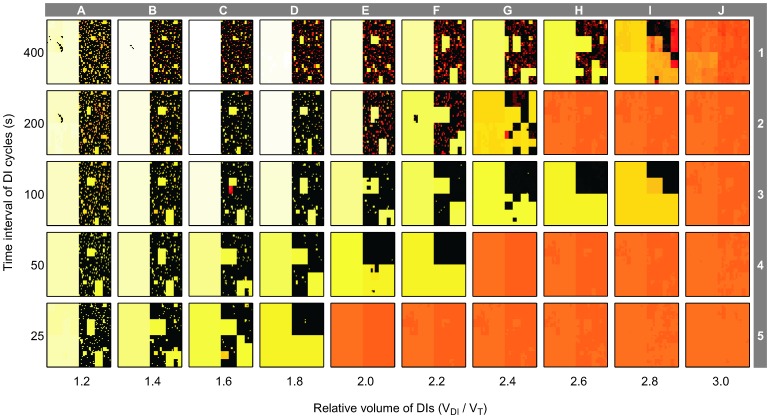
Effects of the relative volume of DIs and the time interval of their cycles on ventilation distribution. The different conditions are organized as a matrix of relative volume of DIs – V_DI_/V_T_ (labeled A–J) and the time interval of DI cycles (labeled 1–5). The color scale of the ventilation maps is identical to that in Fig. 1a (0–1.85).

DI Characteristics affected the emergence of VDefs and resulted in two distinct domains among the conditions of a matrix with linear stepping for the relative volume of DIs and exponential stepping for the time interval of DI cycles (Fig. 4). In one domain, ventilation distribution was homogeneous, which suggests that the volume of DIs and the time interval of DI cycles were sufficient to prevent the emergence of VDefs. For example, at a time interval of DIs cycles of 25 s, V_DI_/V_T_≥2.0 were sufficient to prevent the emergence of VDefs, as the conditions E5-J5 in Fig. 4 illustrates. In the other domain, although the combinations of DI volume and time interval of DI cycle could not prevent VDefs, they affected the pattern and size of VDefs. Close to the boundary between the two domains, VDefs were smaller and more consolidated as the lack of ventilated terminal units inside the VDefs showed, e.g. conditions D5, F4, and I3 in Fig. 4. Also, the general trend of the boundary suggested that there is a synergistic effect of the volume of DIs and the time interval of DI cycles on the emergence of VDefs; e.g. larger volumes and shorter time intervals (i.e. more frequent DIs) being more beneficial.

**Figure 5:**
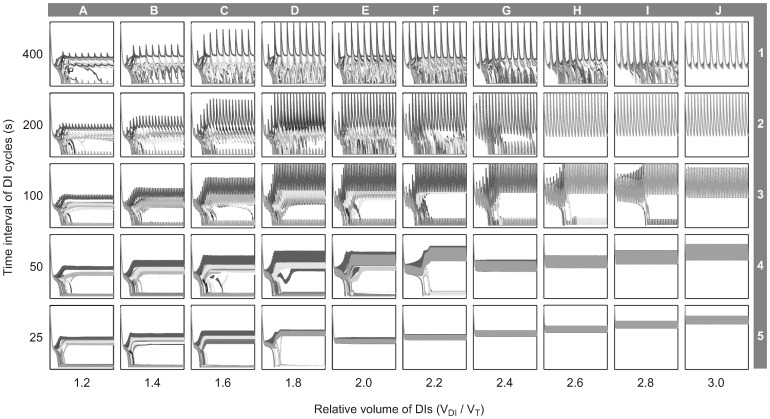
Effects of the relative volume of DIs and the time interval of their cycles on relative airway radii (*r/r_0_*). The different conditions are organized as a matix identical to Fig. 4, and each condition shows 256 randomly selected terminal airways over time (4000 s) using the same x- and y-axes as in Fig. 1c. A set of gray values was used to provide visual distinction among the airways and the differences in airway behavior.

Relative to the stepping of the matrix, the boundary of the domain without VDefs appeared to have an approximately linear trend in the matrix of ventilation maps from V_DI_/V_T_≥2.0 for the time interval of DI cycles of 25 s (condition E5 in Fig. 4) to V_DI_/V_T_≥3.0 for the time interval of 100 s (J3). Surprisingly, V_DI_/V_T_≥2.6 was sufficient to prevent VDefs for the longer time interval of 200 s (H2-J2). But the next longer time interval DI cycles of 400 s required again a larger relative volume of DIs with V_DI_/V_T_≥3.0 (J1) to avoid VDefs. The dynamic behavior of relative airway radii over time for conditions G4-J4 and G5-J5 in Fig. 5 showed that the periodic peak values – caused by periodic dilation – were primarily determined by the relative tidal volume of DIs, while the time interval of DI cycle determined the amplitude of the periodic changes. Interestingly, the conditions H2-J2 had smaller airway radii during the DI cycles–higher constriction–than J3 but no VDefs, which suggests that the higher resistance during the DI causing a higher peak transmural pressure may have prevented the emergence of VDefs in contrast to H3 and I3.

### Conditions without VDefs

In the domain of conditions without VDefs, larger volumes of DIs resulted in an increase in the peak radii during the DI cycle illustrating the volume dependence of airway dilation, e.g. conditions E5-J5 in Fig. 5. That increase suggests a dynamic equilibrium similar to tidal breathing, but with periodic changes induced by the larger relative volume of DIs that cause higher peak transmural pressures at the airway walls and increased airway dilation compared to monotonous breathing without DIs. Also, an increase in the time interval of DI cycles led to larger periodic changes in radii, e.g. conditions J5-J2.

### Conditions with VDefs

**Figure 6:**
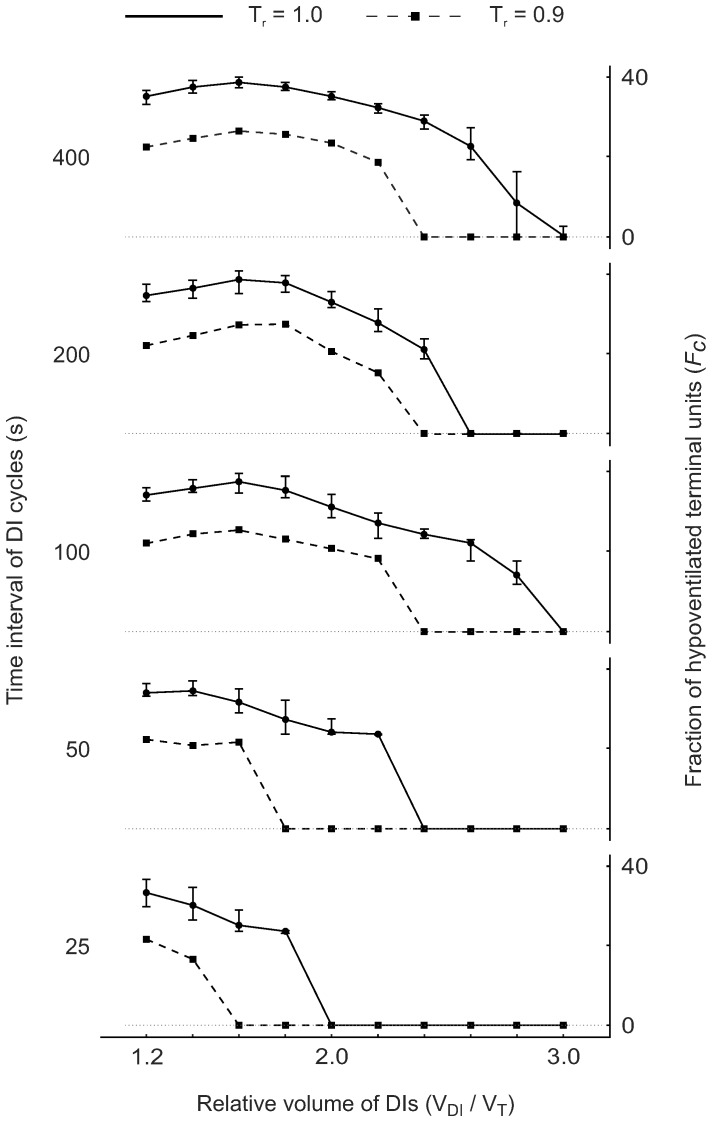
Effects of the relative smooth muscle stimulation (*T_r_*) on mean value of the fraction of closed or hypoventilated terminal units over the last complete DI cycle (*F_c,mean_*) for different conditions, including the relative volume of DIs (V_DI_/V_T_) and the time interval of DI cycles. In addition, effects of 10 different random perturbations on *F_c,mean_* are plotted for the maximum stimulation (*T_r_* = 1.0, solid line), where the error bars show the range, i.e. minimum and maximum values.

In the domain of conditions with VDefs, the individual conditions showed that the interactions between DIs and the heterogeneous bronchoconstriction resulted in more complicated airway behaviors compared to those without VDefs. In general, each DI caused an immediate increase of airway radii in some or all airways followed by a re-narrowing during the time interval between DIs. An increase of the small volume of DIs resulted primarily in greater airway dilation in the least constricted airways, as the increase in peak airway radii during the corresponding DI cycles showed, e.g. condition A2 compared to C2 in Fig. 5. Interestingly, that regional dilation decreased the post-DI resistance (Fig. 3c). In addition, under most of the conditions A1-C5, DIs had only a very small or no effect on severely constricted or closed airways. Interestingly, larger volumes of DIs that dilated some airways to their maximum radii, also affected severely constricted airways as the dynamic changes in airway radii of this group of airways indicated, e.g. condition G2 in Fig. 5. Additionally, larger volumes of DIs were effective in reducing *F_c,mean_* values (Fig. 6). Note that for time intervals of 100, 200, and 400 s, peak values of *F_c,mean_* were reached at V_DI_/V_T_ = 1.6, which coincide with periodic peak radii of some airways slightly below their maximum values (C3-C1 in Fig. 5), while the next higher V_DI_/V_T_ resulted in peak transmural pressures that eventually affected severely constricted airways too, e.g. condition C2 compared to D2. Note that the severely constricted or closed terminal airways that were partially dilated by DIs eventually returned to their original closed state, e.g. conditions F2 and G2 in Fig. 5.

### Level of Smooth Muscle Activation

Effects of the relative airway smooth muscle stimulus on the emergence of VDefs and on *F_c,mean_* showed that the synergistic effect of the relative volume of DIs and the time interval of DI cycles also exists below maximum smooth muscle activation. However, smaller volumes of DIs or longer time intervals of DI cycles were sufficient to prevent the emergence of VDefs at a smooth muscle stimulation of 90% of the maximal stimulation (*T_r_* = 0.9) (Fig. 6). Also, the *F_c,mean_* values were lower for *T_r_* = 0.9 compared to maximal activation of airway smooth muscle (*T_r_* = 1.0); the maximum of *F_c,mean_* was 28.9% for *T_r_* = 0.9 compared to 42.8% for maximum stimulation.

### Random Perturbations

Effects of random perturbations in wall thickness on the emergence of VDefs and on *F_c,mean_* were very small (on the curve *T_r_* = 1.0 in Fig. 6), although the random perturbations had affected the location and shapes of VDefs in ventilation maps of conditions with VDefs. The deviations of the individual values of *F_c,mean_* for the 10 random realizations were in average plus 2.6% and minus 2.3% relative to the mean of each condition. The emergence of VDefs was not affected by the random perturbations except for one out of the 10 perturbations at V_DI_/V_T_ = 2.8 with the time interval of DI cycles of 400 s, which did not trigger the emergence of VDefs (Fig. 6).

### Speed of Smooth Muscle Shortening

**Figure 7:**
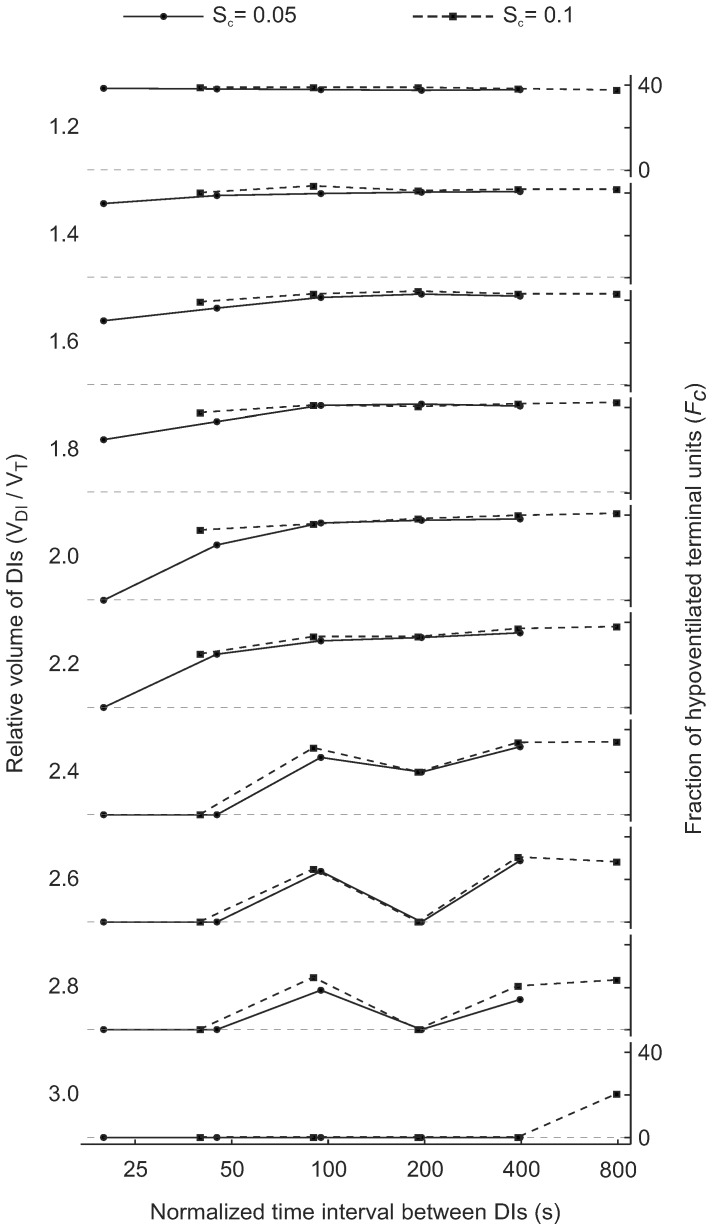
Effects of the speed index of constriction (*S_c_*) on *F_c,mean_* for different conditions, including the relative volume of DIs (V_DI_/V_T_) and the time interval between DIs normalized by *S_c_*. Note that doubling the normal speed index of constriction (dashed vs. solid lines) appears to result in very similar behaviors at the time interval between DIs normalized by *S_c_*, suggesting that the ratio of the two parameters determines the airway behavior.

The speed index of constriction (*S_c_*) had a substantial effect on airway behavior. However, we found that the differences virtually disappeared for the time interval between DIs normalized by *S_c_* (Fig. 7), which demonstrates that the efficiency of a time interval between two consecutive DIs depends of the time constant of smooth muscle re-narrowing after each DI, and that this relationship can be described by a single parameter such as the normalized time interval between DIs. In addition, the effect of increased *S_c_* on the fraction of hypoventilated terminal units illustrates that if airway remodeling leads to faster smooth muscle constriction it would require shorter time intervals of DI cycles to prevent the emergence of VDefs. For the same time intervals of DI cycles the risk for the emergence of VDefs would be increased.

### Extended Time Intervals and Larger Volumes of DIs

**Figure 8:**
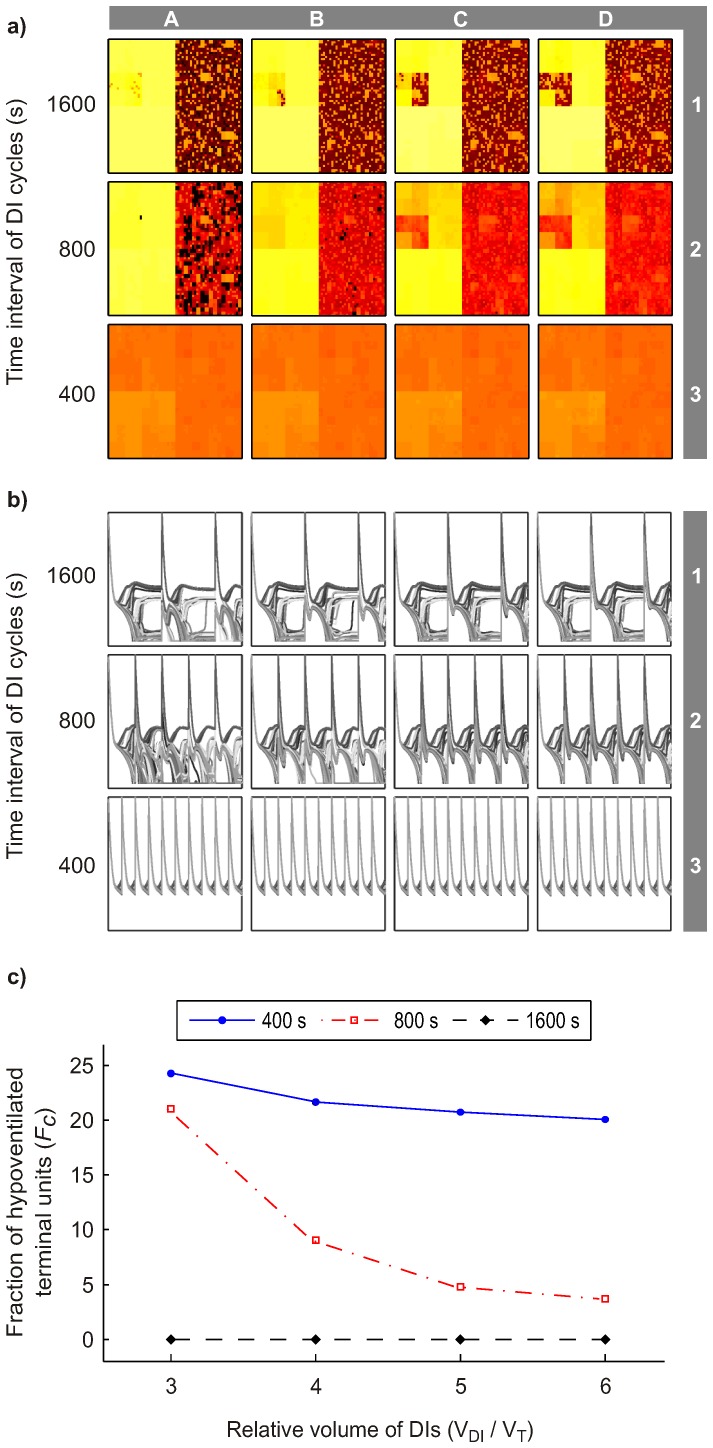
Effects of longer time intervals of DI cycles and larger DI volumes than in Figs. 4 and 5. Ventilation distribution maps (a) and relative airway radii (*r/r_0_*) (b) of 256 randomly selected terminal airways over time (4000 s) for different conditions using the same scales as in Figs. 1, 4 and 5. The corresponding *F_c,mean_* values of the same conditions are plotted in (c).

**Figure 9:**
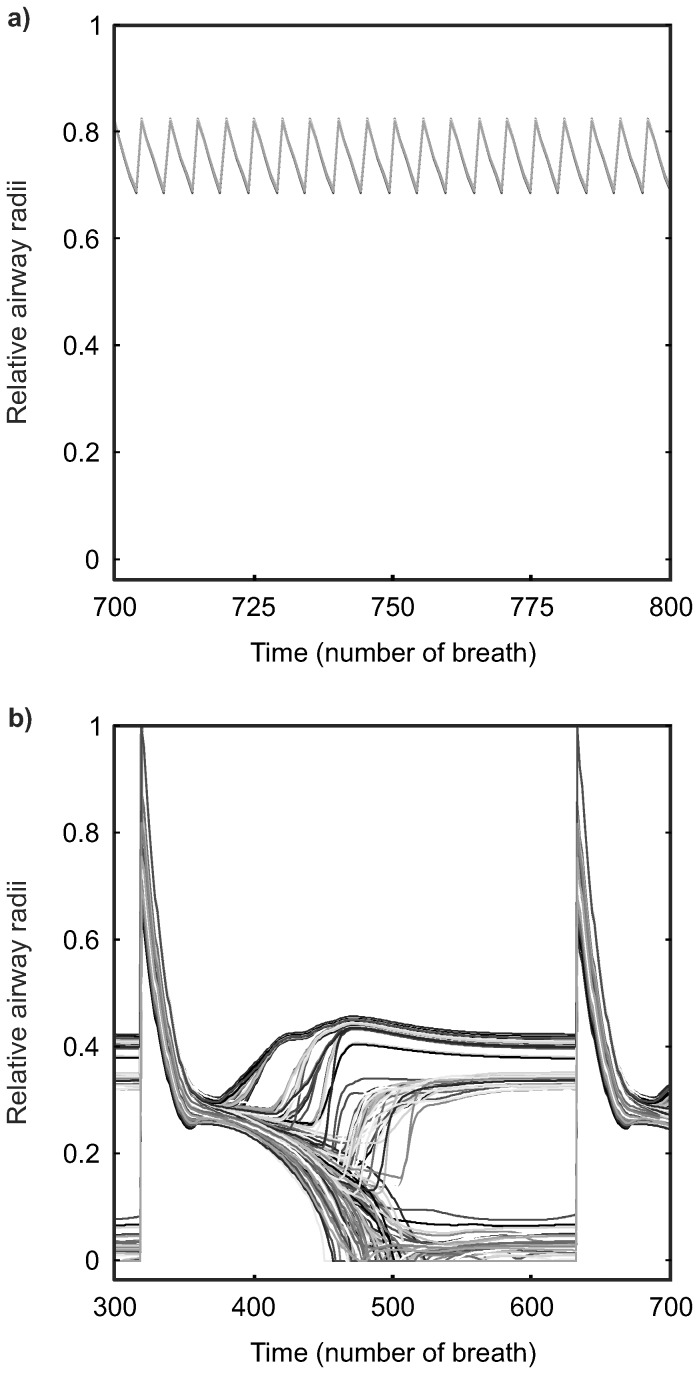
Details of dynamic changes in airway radii in response to DIs at two selected conditions. The two examples emphasize conceptually relevant details, such as, (a) periodic changes in airway radii under conditions without VDefs (condition J5 in Fig. 5) and (b) bronchodilation followed by re-emergence of severe bronchoconstriction and VDefs at very long time intervals of DI cycles (condition D1 in Fig. 8b), which may have been too small in previous figures. Note that in (a), DIs caused some dilation followed by re-narrowing so that airway radii change periodically. The behavior of all 256 randomly selected terminal airways is uniform so that it appears to be a single airway. In (b), the larger volume of DIs and the longer time intervals between them caused substantial dilation of all airways followed by not only re-narrowing of airways, but also re-emergence of heterogeneous bronchoconstriction and VDefs.

Effects of extended time intervals of DI cycles and larger volumes of DI on heterogeneous bronchoconstriction were different from those of shorter time intervals (≤400 s) and smaller volumes (V_DI_/V_T_≤3) (Figs. 8a and b compared to Figs. 4 and 5, respectively). The time interval of DI cycles of 400 s resulted in long periods of monotonous breathing between DIs that allowed the emergence of some degree of heterogeneity in bronchoconstriction (conditions A3-D3 in Figs. 8a and b). However, sufficiently large volumes of DIs (V_DI_/V_T_≥3) periodically dilated all airways to their maximum radii, which prevented the emergence of VDefs. For even longer time intervals of DI cycles (1600 s), the time between the periodic dilation of all airways was so long that even DIs reaching total lung capacity (V_DI_/V_T_ = 6) could not prevent the emergence of VDefs (conditions A1-D1 in Figs. 8a and b). In contrast to conditions without VDefs where more frequent DIs caused periodic homogeneous dilation, e.g. Fig. 9a, the dynamic changes in relative airway radii at a condition with VDefs showed that less frequent DIs with large volumes were able to dilate the most constricted airways and to resolve VDefs, but such long time interval between DIs allowed airway re-narrowing to a degree that VDefs emerged (Fig. 9b). This periodic airway behavior converged to virtually identical cycles with major changes in the heterogeneity of constriction during each cycle. Note that a time interval of DI cycles of 1600 s corresponds to periods of more than 26 minutes without a DI.

## Discussion

The main findings of our study are that: 1) the normalized time interval between DIs is a parameter that describes airway re-narrowing between DI in respect to speed or time constant of smooth muscle constriction, 2) large volume of DIs separated by long time intervals can dilate airways and transiently resolve VDefs, but VDefs re-emerge before the next DI, 3) DIs can interact with the heterogeneous bronchoconstriction, that is associated with VDefs, and cause transient dynamics in airway radii that may include constriction and dilation occuring in different airways, 4) the decrease in airway resistance in response to a DI as well as the trends prior and after each DI are similar in the computational model and experimental data, 5) the relative volume of DIs (V_DI_/V_T_) and the time interval of DI cycles have a synergistic effect on bronchoconstriction and the emergence of VDefs suggesting that differences in these parameters can have a major affect on the status of patients and the results of studies, 6) at lower levels of airway smooth muscle activation, smaller DI volumes and longer time intervals between them are sufficient to prevent VDefs suggesting that the efficiency of DIs depends on the challenge of the smooth muscle and potentially other factors, and 7) differences in the random realizations used to perturb the symmetry in the model change the topography of the VDefs, but not the fractions of hypoventilated units at a given condition,

In the present study, we showed results from our integrative model of bronchoconstriction for breathing patterns with and without DIs. The computational model allowed us to evaluate current paradigms of bronchodilation and bronchoprotection in response to DIs in a bronchial tree where interactions among airways may result in the emergence of complex behavior such as self-organized patterns of bronchoconstriction. Such phenomena of complex behavior critically rely on mathematical and computational modeling, mainly because the number of interactions among the elements within the network is too large for experimental studies as well as for analytical solutions of the network's behavior. The computational network model can help to close the gap between experimental accessibility of dynamic airway behavior within a bronchial tree and theoretical knowledge of single airway behavior, which is the network element. Understanding such interactions could be highly relevant to aid the interpretation of experimental observations.

A Comparison of the Model and Experimental Data on the bronchodilatory effect of a DI on airway resistance from Hulme et al. showed excellent agreements in multiple aspects. In fact, we had not expected that changes in airway resistance caused by re-narrowing prior and after a DI may have resembled experimental data so closely. For comparison purposes, we chose to use the mean of end-inspiratory and end-expiratory resistance of each breath of the experimental data, which had the limitation that the very low resistance at the high end-inspiratory lung volume during the DI in the experimental data led to a much lower mean resistance of that DI compared to tidal breathing. Also, the example of a change in airway resistance in [7] did not include details of the specific DI, or subject or group specific parameters that would allow adjustments of the model. Given these limitations, it was necessary to select one condition of the simulations with a sufficiently long interval of DI cycles and a similar response in *R*_Post-DI_/*R*_Pre-DI_. The selected DI characteristics showed changes in airway resistance over time prior and after the DI that were surprisingly similar to the experimental data suggesting that this is evidence for valid characteristic predictions of the computational model, including interactions between DIs and self-organized bronchoconstriction in an airway tree.

The volume of DIs affects the bronchodilatory response of airways [9], and the relative decrease in the resistance of the respiratory system [7]. To compare the model's predictions with experimental data, we aimed to demonstrate similarities in the normalized response *R*_Post-DI_^*^. The normalization eliminates differences that originate from a lack of details about experimental conditions, and of model parameters matching individual subjects. There are two specific aspects in the comparison of normalized responses that showed the similarity in the characteristic behaviors: 1) above the DI volume where *R*_Post-DI_^*^ started to fall–the onset of the response–, larger volumes of DIs resulted in lower *R*_Post-DI_^*^, and 2) above the DI volume at which *R*_Post-DI_^*^ reached a minimum, larger volumes of DIs resulted in *R*_Post-DI_^*^ similar to the minimum, which suggests a plateau in the response. The *R*_Post-DI_ values without normalization showed lower model predictions compared to the experimental data [7] at the same fraction of the inspiratory capacity. That illustrates a difference that can be explained by a lower resistance of the unconstricted airway tree in the model compared to the subjects in the study. Subject specific model parameters would reduce that difference. Another source of the differences between model predictions and experimental data may be differences in the breathing conditions, such as the tidal volume of the subjects. For example, Duggan et al. noted that some of their subjects had difficulties to perform a DI at 60% of TLC because that was approximately their end-inspiratory volume [8]. Also, the effect of DI volume on *R*_Pre-DI_ in our findings demonstrates that the breathing conditions before a DI may affect the magnitude of the response in *R*_Post-DI_/*R*_Pre-DI_ in experimental studies. Persistent airway closure and liquid plugs could also cause differences in the response to DIs. If asthmatics had such persistent closure of airways inside of VDefs in combination with very dilated airways outside of VDefs that could potentially explain the difference in the response to DIs between asthmatics and non-asthmatics reported by Hulme et al. [7]. We like to emphasize that factors, such as the status of lung inflation or end-inspiratory volume, may affect the responses to DIs, although they are rarely reported.

The Synergistic Effect of the Volume of DIs and the Time Interval of DI Cycles on the degree of bronchoconstriction and on the emergence of VDefs can be explained by not only the dynamics of airway response to a transient stretch, but also the interactions among airways and the branching structure of the bronchial tree. Dynamic changes of airflows, pressures, and parenchymal inflation during normal breathing cycle or a DI lead to changes in the peak transmural pressures of the individual airways. The local interactions between the peak transmural pressure and the virtual pressure caused by smooth muscle tension determine the airway radii during bronchoconstriction. As shown in Fig. 5, during a given DI cycle, the larger volume of DI cycles compared to the normal tidal volumes between DIs causes higher peak transmural pressures, which consequently result in rapid dilation followed by re-narrowing of airways. At conditions without VDefs, this leads to periodic changes in airway caliber relative to peak airway radii that are affected by the relative volume of DIs. At conditions with VDefs, heterogeneous bronchoconstriction affects the distribution of the larger volume of DIs, which can result in heterogeneous peak transmural pressures, and may cause heterogeneous airway responses to DIs.

The Time Constant For Airway Re-narrowing After a DI, estimated using measurements of resistance of the respiratory system, was found to be substantially shorter in asthmatics compared to non-asthmatics: 12 vs. 35 s [31]. It is unclear if that difference is caused by a change in the speed of airway smooth muscle shortening or if other factors may be responsible for that difference. Nonetheless, understanding the potential effect of a higher speed in airway smooth muscle shortening is relevant for the interpretation of experimental data. We showed that the speed index of smooth muscle shortening can alter the dynamics of airway behavior as well as the emergence of VDefs. Surprisingly, despite the complex interactions among airways during bronchoconstriction, we found that the effect can be explained by a single parameter: the normalized time interval between DIs defined as the time interval between DIs times the actual speed index *S*_c_ divided by the normal speed index. Note that an increased speed index of constriction converts the actual time interval between DIs into longer time interval on the normalized scale as if a patient had less frequent DIs. This means that in case asthmatics have a higher speed of smooth muscle constriction, more frequent DIs may be needed to prevent the emergence of VDefs or their level of bronchoconstriction would be worse, as differences between longer and shorter time intervals of DI cycles in our study suggested.

Airway Smooth Muscle Activation involves multiple interdependent factors including the concentration of inhaled agonists, its deposition, and the dose response of the smooth muscle. Additional sources that can contribute to activation of smooth muscle in vivo include the vagus nerve and indirectly airway inflammation. Lavoie et al [32] showed that agonist concentration affecting the level of bronchoconstriction had an effect on the bronchodilatory effect of tidal breathing. The comparison between *T_r_* = 0.9 and 1.0 in our study provides insights into differences in airway behavior that could correspond either to similar differences in agonists concentration, or a difference between hyperresponsive and normal airways exposed to the same agonist concentration. The substantially lower *F_c,mean_* and the larger number of conditions without DIs at the lower *T_r_* suggest that smaller volumes of DIs and longer time intervals of DI cycles were sufficient to prevent the emergence of VDefs. These results of more efficient bronchodilation at lower smooth muscle activation are consistent with the findings of Lavoie et al [32] for tidal breathing.

Different Random Perturbations were used to quantify the variability that the random variation in airway wall thickness, although small, may cause in *F_c,mean_* at different DI conditions. In contrast to random variations, computers make numerical errors that are deterministic so that identical pathways are affected by the same error and the unstable equilibrium of a functionally and structurally symmetric tree remains unperturbed. The feedback-driven differentiation in our computational model of bronchoconstriction is deterministic and reproducible, but relies on a perturbation of the unstable equilibrium. The important aspect of our results is that the random realizations led to major variations in the location of VDefs, but had only a small effect on the functional parameters, as the relative small ranges of *F_c,mean_* values indicated.

Very long Time Intervals of DI Cycles were sufficient for airway radii to reach a relative stable state of constriction prior to the following DI. This shows that the bronchodilatory effect of DIs was not persistent, which is in fact related to airway narrowing during periods of withholding of DIs in experimental studies. Such withholding of DIs for an extended period prior to bronchostimulation results in more severe response compared to normal breathing with spontaneous DIs, which is commonly described as a bronchoprotective effect of DIs. Moore et al [33] reported that withholding of DIs during a methacholine challenge resulted in a greater change in FEV_1_, suggesting more severe bronchoconstriction. Kapsali et al [26] demonstrated that a single-dose methacholine challenge after abstaining from DIs for 20 minutes caused in non-asthmatics a change in FEV_1_ very similar to asthmatics, while repeating the same protocol but taking two or five DIs prior to the challenge resulted in substantially smaller changes in FEV_1_ in non-asthmatics, which suggests a bronchoprotective effect. Similarly, King et al [34] showed that DI avoidance during a protocol with multiple challenges resulted in substantially larger changes in FEV_1_. In our study, the long periods of monotonous breathing between DIs (over 26 min for the longest interval of DI cycles) occurred under conditions of constant smooth muscle activation, which is different from DI avoidance prior or during a bronchial challenge. However, baseline smooth muscle tone [35], that corresponds to *T_r_* = 0.6 in our model [36] leads to some degree of airway narrowing that makes airway radii susceptible to bronchodilation by DIs. Similar to airway re-narrowing during the time interval between DIs, DI avoidance for an extended period prior and during a bronchial challenge may increase airway narrowing, and as a result, an inhaled agonist increasing the activation of smooth muscle relative to baseline tone may worsen bronchoconstriction to a level that it triggers the emergence of VDefs. Multiple DIs immediately before a bronchial challenge may maximally dilate the airways so that a less constricted state can be maintained. In fact, Kapsali et al [26] showed that five DIs to TLC immediately before a single-dose methacholine challenge could completely reverse the condition that 20 minutes of DI avoidance created, while two DIs resulted only in partial reversal. Overall, the sensitivity to DIs prior to an agonist inhalation may suggest that breathing patterns with less frequent and smaller volumes of DIs could increase the chances of the emergence of VDefs in asthmatic patients.

Limitations of Our Simulation Study are important to consider for comparing them with other experimental or computational studies as well as for the application of our results. The study assumed breathing patterns with DIs of defined volumes and time intervals between them. In patients and subjects, these parameters show some natural variability. However, in order to explore quantitatively the basic system's response to DIs, we purposefully kept the breathing rate, DI volume and time interval of DI cycles constant. Future studies could evaluate the effects of the natural variability in tidal volume, respiratory rate, and DI characteristic. Other parameters, such as asymmetry of airway tree branching or thickening of airway walls due to inflammation, are also likely to affect the airway response to DIs, but they require a separate set of simulations. Another potential source of differences may be that the model does not include effects of liquid bridges or mucus plugs, which may require higher pressures for airway reopening and shift the onset of airway responses to higher volumes of DIs. Finally, it should be noted that our computational and mathematical model is constructed based on physical and mechanical properties of the respiratory system, and some of the parameters could only be estimated from animal studies or ex-vivo measurements. In case new studies produce different parameter values, it is likely that quantitative aspects of the model predictions may change, but the general characteristics of our findings resulting from the mechanistic interactions between the system components can be expected to remain similar.

In Summary, we demonstrated bronchodilatory effects of DIs in our integrative model of bronchoconstriction that were similar to experimental measurements. Also, we found that the bronchodilatory effect of DIs did not always reach all airways after VDefs emerged. In fact, interactions between DIs and self-organized heterogeneous bronchoconstriction associated with VDefs can result in major difference in regional airway responses including substantial dilation only in airways outside of VDefs. Differences in VDefs among varying conditions suggest that there is a synergistic effect between the volume and frequency of DIs on their ability to prevent the emergence of VDefs. Evidence from both experimental results and computational modeling suggest that DIs during spontaneous breathing are highly relevant for preventing bronchoconstriction. However, more detailed measurements of DIs, including their volume and frequency, will be required to investigate if differences in DI characteristics may directly contribute to differences in airway behavior between asthmatics and non-asthmatics. Since complex behavior in asthma is not directly accessible, computational modeling of airway responses in a bronchial tree as well as the interactions between heterogeneous bronchoconstriction and DIs, is essential for interpreting experimental results and gaining a better mechanistic understanding of airway behavior in asthmatics.
